# Supplementary material for: In silico fragment-based discovery of CIB1-directed anti-tumor agents by FRASE-bot
Source: Nat Commun. 2024 Jul 2;15:5564. doi: 10.1038/s41467-024-49892-9 (PMC11219766; doi:10.1038/s41467-024-49892-9)

In silico Fragment-based Discovery of CIB1-directed Anti-Tumor Agents by FRASE-bot

Yi An[1], Jiwoong Lim[1], Marta Glavatskikh[1], Xiaowen Wang[1,2], Jacqueline Norris-Drouin, P. Brian Hardy[1], Tina M. Leisner[1], Kenneth H. Pearce[1*], and Dmitri Kireev[1,2,3*]

[1]Center for Integrative Chemical Biology and Drug Discovery, UNC Eshelman School of Pharmacy, University of North Carolina, Chapel Hill, NC 27513

[2]Chemistry department, University of Missouri, Columbia, Columbia, MO, 65211

[3]Lead contact

[*]Correspondence: khpearce@unc.edu and dmitri.kireev@unc.edu

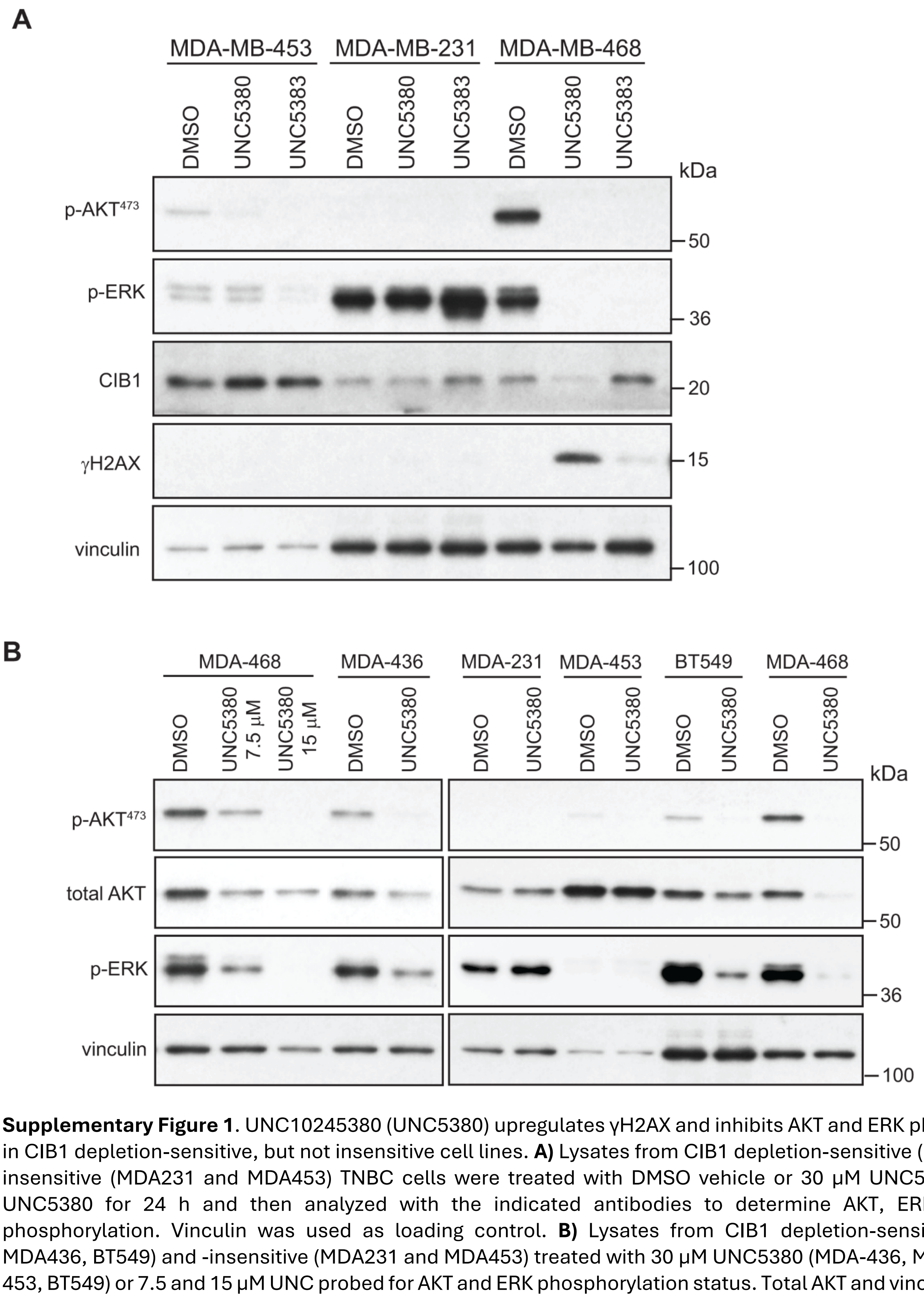

Supplementary Figure 1. UNC10245380 (UNC5380) upregulates γH2AX and inhibits AKT and ERK phosphorylation in CIB1 depletion-sensitive, but not insensitive cell lines. **A)** Lysates from CIB1 depletion-sensitive (MDA468) and -insensitive (MDA231 and MDA453) TNBC cells were treated with DMSO vehicle or 30 µM UNC5383* or 15 µM UNC5380 for 24 h and then analyzed with the indicated antibodies to determine AKT, ERK, and γH2AX phosphorylation. Vinculin was used as loading control. **B)** Lysates from CIB1 depletion-sensitive (MDA468, MDA436, BT549) and -insensitive (MDA231 and MDA453) treated with 30 µM UNC5380 (MDA-436, MDA-321, MDA-453, BT549) or 7.5 and 15 µM UNC probed for AKT and ERK phosphorylation status. Total AKT and vinculin were used as loading controls. Western blots are representative of 3 independent experiments (MDA-468 and MDA-231) and 2 independent experiments (MDA-436, MDA-453, BT549)

*UNC5383 is a compound unrelated to this manuscript.

Original gel images for Supplementary Fig. 1A

10 seconds exposure

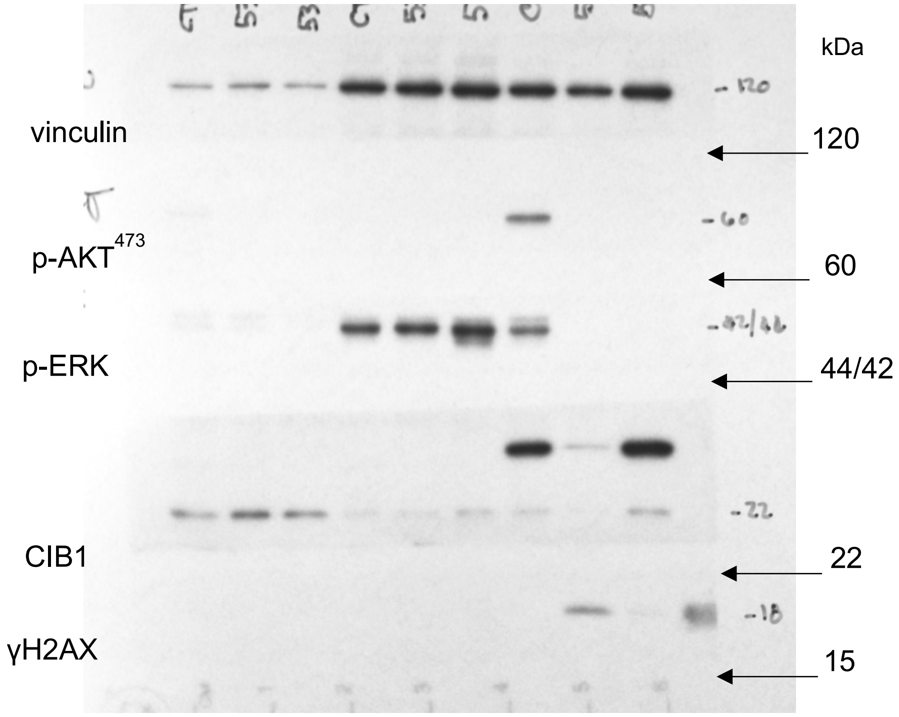

30 seconds exposure

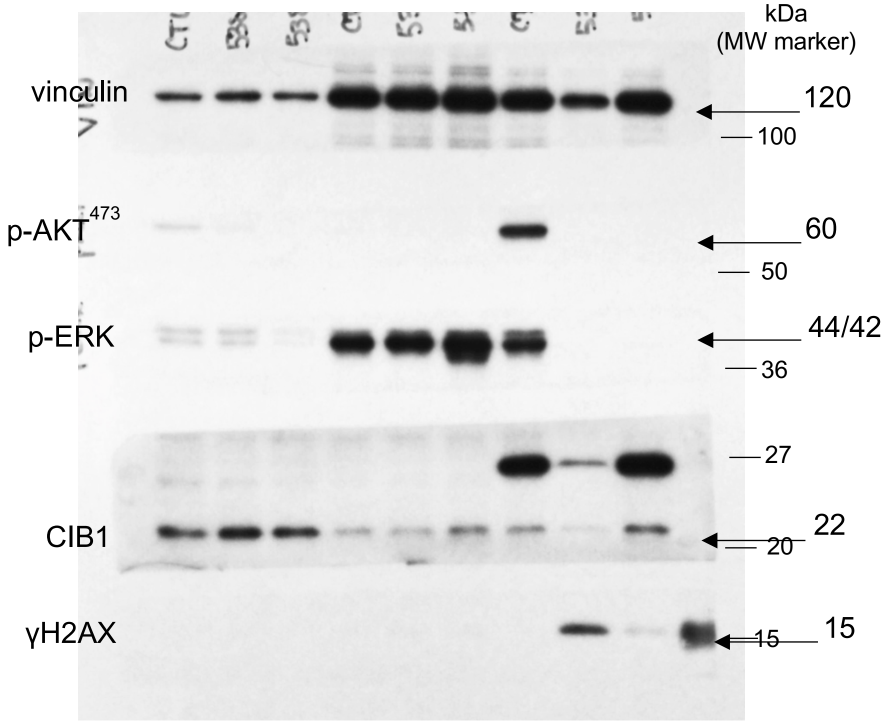

Original gel images for Supplementary Fig. 1B

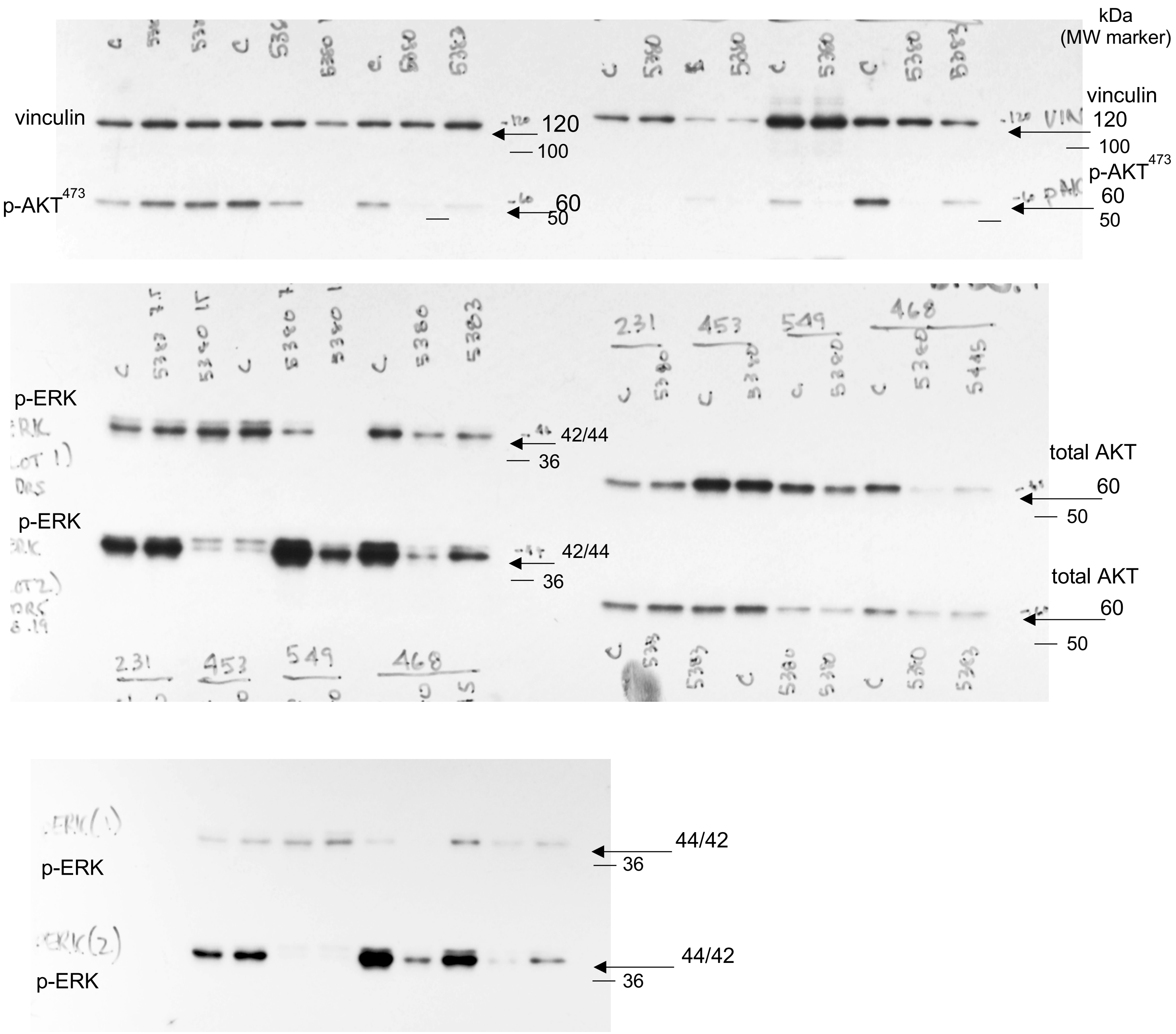

Supplement: Supplementary file 1 — Supplementary Information [file 41467_2024_49892_MOESM1_ESM.pdf]
