# Supplementary material for: In silico fragment-based discovery of CIB1-directed anti-tumor agents by FRASE-bot
Source: Nat Commun. 2024 Jul 2;15:5564. doi: 10.1038/s41467-024-49892-9 (PMC11219766; doi:10.1038/s41467-024-49892-9)
Supplement: Supplementary file 3 — Description of Additional Supplementary Files [file 41467_2024_49892_MOESM3_ESM.pdf]

Description of Additional Supplementary Files

File Name: Supplementary Data 1

Description: TR-FRET data for 56 compounds selected by virtual screening, as well as for 24 compounds resulting from the SAR-by-catalog study.
